# Supplementary material for: Spreading of Aggregated α-Synuclein in Sagittal Organotypic Mouse Brain Slices
Source: Biomolecules. 2022 Jan 19;12(2):163. doi: 10.3390/biom12020163 (PMC8961638; doi:10.3390/biom12020163)
Supplement: Supplementary file 1 [file biomolecules-12-00163-s001.zip › biomolecules-1536685-supplementary.pdf]

Supplementary Material

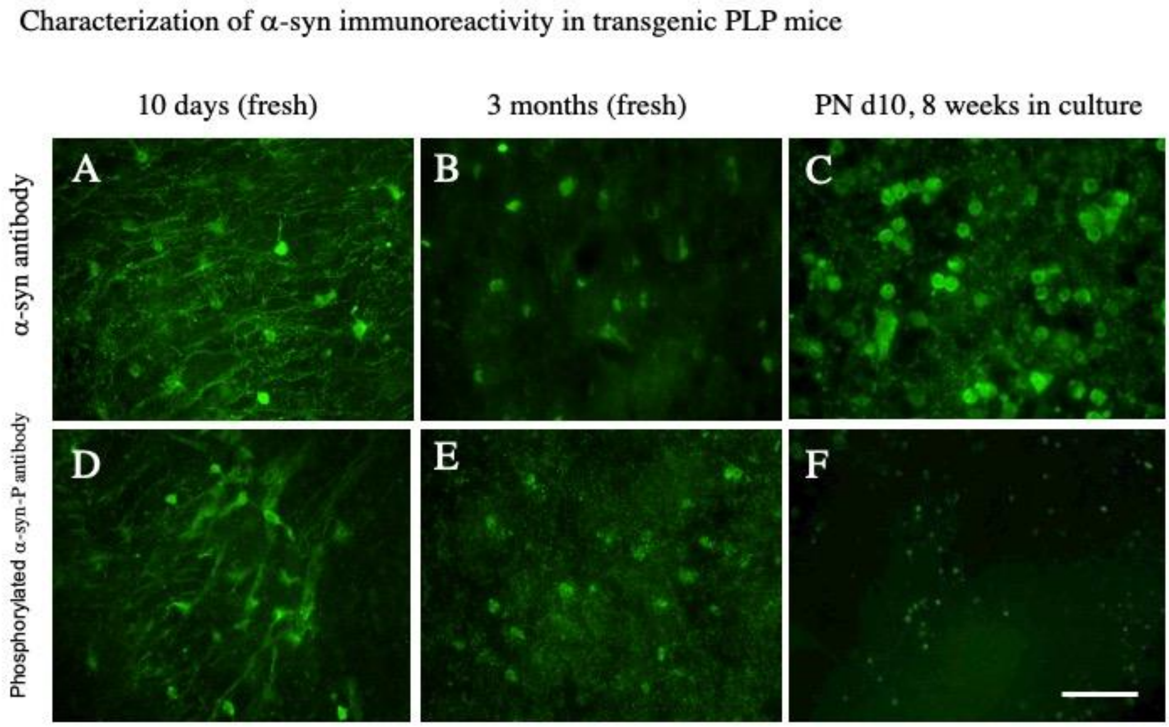

Figure S1. *Characterization of the immunostainings using α-syn (**A**–**C**) or α-syn-P (D–F) antibodies.* Staining was performed from paraformaldehyde-fixed brains taken from 10-day-old transgenic mice (**A**,**D**) or 3-month-old transgenic mice (**B**,**E**), or slices taken from 10-day-old mice and cultured for 8 weeks (**C**,**F**). Note a strong α-syn and α-syn-P-like immunoreactivity in 10-day-old mice (**A**,**D**), which markedly decreased in 3-month-old mice (**B**,**E**). In brain slices cultured for 8 weeks (taken from 10-day-old transgenic mice), a strong α-syn immunoreactivity was seen but nearly no α-syn-P staining. Scale bar, F = 280 μm (**A**,**D**) and 420 (**B**,**C**,**E**,**F**).
